# Effect of pioglitazone treatment on behavioral symptoms in autistic children

**DOI:** 10.1186/1742-2094-4-3

**Published:** 2007-01-05

**Authors:** Marvin Boris, Claudia C Kaiser, Allan Goldblatt, Michael W Elice, Stephen M Edelson, James B Adams, Douglas L Feinstein

**Affiliations:** 1 77 Froehlich Farm Blvd Woodbury, New York 11797, USA; 2 Department of Anesthesiology, University of Illinois, Chicago, IL, 60612, USA; 3 Autism Research Institute, 4182 Adams Ave, San Diego, CA 92116, USA; 4 Arizona State University, PO Box 876006, Tempe, AZ 85287-6006, USA

## Abstract

**Introduction:**

Autism is complex neuro-developmental disorder which has a symptomatic diagnosis in patients characterized by disorders in language/communication, behavior, and social interactions. The exact causes for autism are largely unknown, but is has been speculated that immune and inflammatory responses, particularly those of Th2 type, may be involved. Thiazolidinediones (TZDs) are agonists of the peroxisome proliferator activated receptor gamma (PPARγ), a nuclear hormone receptor which modulates insulin sensitivity, and have been shown to induce apoptosis in activated T-lymphocytes and exert anti-inflammatory effects in glial cells. The TZD pioglitazone (Actos) is an FDA-approved PPARγ agonist used to treat type 2 diabetes, with a good safety profile, currently being tested in clinical trials of other neurological diseases including AD and MS. We therefore tested the safety and therapeutic potential of oral pioglitazone in a small cohort of children with diagnosed autism.

**Case description:**

The rationale and risks of taking pioglitazone were explained to the parents, consent was obtained, and treatment was initiated at either 30 or 60 mg per day p.o. A total of 25 children (average age 7.9 ± 0.7 year old) were enrolled. Safety was assessed by measurements of metabolic profiles and blood pressure; effects on behavioral symptoms were assessed by the Aberrant Behavior Checklist (ABC), which measures hyperactivity, inappropriate speech, irritability, lethargy, and stereotypy, done at baseline and after 3–4 months of treatment.

**Discussion and evaluation:**

In a small cohort of autistic children, daily treatment with 30 or 60 mg p.o. pioglitazone for 3–4 months induced apparent clinical improvement without adverse events. There were no adverse effects noted and behavioral measurements revealed a significant decrease in 4 out of 5 subcategories (irritability, lethargy, stereotypy, and hyperactivity). Improved behaviors were inversely correlated with patient age, indicating stronger effects on the younger patients.

**Conclusion:**

Pioglitazone should be considered for further testing of therapeutic potential in autistic patients.

## Introduction

Autism, the most common of the group of disorders collectively referred to as Autism Spectrum Disorders (ASD), is a complex neurological disease of unknown etiology. The incidence of autism is estimated to be 1 per 166 [1] with a male to female ratio of 4:1. Autism has been found throughout the world in families of all racial, ethnic and social backgrounds. Although accumulating evidence suggests that genetic, environmental, inflammatory, immunological, and metabolic factors play a prominent role in this disease [2-7], the precise causes remain to be determined.

Altered immune responses in children with ASD are well documented. Autoimmune disorders of thyroiditis, colitis, myelin basic protein autoantibodies, and diabetes are prevalent in children with ASD. Stubbs (1976) published that 5 of 13 autistic children had no detectable rubella antibodies despite prior immunization [7]. An additional study showed peripheral mononuclear cells had a decreased proliferative response to mitogenic stimulation compared to normal children [8]. These findings of abnormal T-lymphocyte function have been replicated by other investigators [9,10]. Inflammatory responses in ASD have also been reported to occur in brain, for example neuroinflammatory processes involving both microglia and astroglia were found on post mortem examination in autistic children with elevated cytokine levels in the cerebral spinal fluid [11,12]. Children with ASD have increased cytokines of Th2 and Th1 arms of the immune response with Th2 predominant without an increase in IL10 [13].

Peroxisome proliferator-activated receptor gamma (PPARγ) is a nuclear hormone receptor originally characterized by its ability to regulate adipocyte differentiation and gene transcription [14]. PPARγ agonists include fatty acids, non-steroidal anti-inflammatory drugs (NSAIDs), the natural compound 15-deoxy12,14-prostaglandin-J2 (PGJ2), and members of the class of synthetic drugs termed thiazolidinediones (TZDs) which include pioglitazone (Actos) and rosiglitazone (Avandia). TZDs were originally designed as anti-diabetic drugs due to their insulin sensitizing effects, and several are now in clinical use. In addition to insulin sensitizing effects, TZDs also exert anti-inflammatory effects on a variety of cell types, and for this reason some are being considered for treatment of inflammatory diseases including artherosclerosis [15], psoriasis [16,17], and inflammatory bowel disease [18-21]. TZDs also reduce inflammatory activation of brain glial cells, and increase metabolic activities in glial cells which can lead to increased glucose uptake, lactate production, and mitochondrial function [22,23]. Furthermore, pioglitazone can cross the BBB, [24] suggesting possible direct effects on brain physiology, which could positively influence possible abnormalities in regional brain glucose utilization [25] or dysregulation of functional activity [26] as reported to occur ASD.

The safety and efficacy of pioglitazone has been established by clinical studies worldwide [27,28] and since FDA approval, pioglitazone has been prescribed to several million patients. The adverse events associated with TZDs including pioglitazone are generally mild and transient, and those effects returned to baseline upon withdrawal from, or completion of the studies. Two recent studies for the treatment of diabetes in adolescents point to a good safety profile for Actos in younger populations [29,30]. Studies with PPARγ drugs in animal models of neurological conditions have led to clinical testing of these drugs in Alzheimer's disease (AD) and multiple sclerosis (MS) [31,32]. These properties of PPARγ agonists make them promising candidates for a therapeutic approach to influence the clinical course of ASD. In this report we discuss initial findings using pioglitazone to treat children with autism, which provides the rationale for design of larger clinical trials.

## Case description

### Population

The autistic children all were patients of Marvin Boris, MD, Allan Goldblatt, PA, and Michael Elice, MD. Twenty-five children and adolescents participated in this study. The mean age was 7.9 ± 3.5 years, with a range from 3 to 17 years. There were 22 males and 3 females. All of the participants received an independent diagnosis of Autism Spectrums Disorder (ASD) from an independent clinician and/or agency. None of the children had diagnosed Asperger's Disorder (a mild variant of ASD with higher social functioning) or PDDNOS (Pervasive Developmental Disorder – Not Otherwise Specified, a condition with social or behavioral impairments but which do not meet the DSM-IV criteria for ASD). The diagnosis of autism was initially established by a board certified pediatric neurologist, developmental pediatrician, or psychiatrist with experience in ASD. In addition, at the first visit to the offices of the treating physician, the child had to meet the DSM-IV checklist criteria for ASD. All the children had been receiving behavioral and educational therapies. These included speech, occupational, and physical therapy, applied behavioral analysis, and auditory integration therapy. The children had also received various biomedical interventions for at least one year. These included dairy and gluten free diet, metabolic treatment with supplements to known deficiencies such as MTHFR (methylenetetrahydrofolate reductase), treatment with intravenous gamma globulin or secretin, vitamin supplementation, and heavy metal chelation. The children who responded poorly (no noticeable improvements in cognitive, social, behavior, or language skills) for at least one year to biomedical, behavioral, or educational therapies were selected to be treated with pioglitazone as part of the routine health care treatment, based on papers suggesting that ASD includes an auto-immune or inflammatory component [33,34], and that pioglitazone can reduce T-cell activation and Th2-type cytokine production, both implicated in ASD [35-38]. The rationale and risks of taking pioglitazone were explained to the parents, and parental written consents were obtained for all participants. A retrospective review of their personal medical records was approved by the Internal Review Board of Arizona State University.

#### Comorbities

The autistic population has well-known auto-immunne comorbidities. In this group of autistic children, 7/25 (28%) had thyroiditis, 8/25 (32%) had colitis, 8/25 (32%) had PANDAS (Pediatric acquired neurological disorder associated with streptococcus), 20/25 (80%) had allergic diseases, and 7/25 (28%) were positive for serum antibodies to myelin basic protein. In addition 2/25 had seizures prior to being treated with pioglitazone.

### Treatment

Children were prescribed pioglitazone either 30 mg per day, p.o. for ages 3–5 years old; or 60 mg per day for ages 6–17 years old. These children were followed with monthly complete blood counts, glucose and insulin levels, and serum metabolic assays.

### Analysis

The participants' parents completed the Aberrant Behavior Checklist (ABC) prior to the administration of pioglitazone and then at a follow-up assessment, 12 or 16 weeks later. There are five subscales on the ABC, consisting of 58 questions. The subscales are: hyperactivity, inappropriate speech, irritability, lethargy, and stereotypy. Each question was rated on a 4-point scale: 0 = 'not a problem,' 1 = 'the behavior is a problem but slight in degree,' 2 = 'the problem is moderately serious,' and 3 = 'the problem is severe in degree.' 'The ABC has been shown to be a valid and reliable procedure to evaluate treatment efficacy [39-41]. Each of the five subscales was analyzed using paired t-tests. The relationship between age and amount of behavior change was examined using Pearson product correlations

### Outcomes

There were no significant abnormalities observed in standard blood analyses in the group of 25 autistic children treated with pioglitazone for up to 4 months (Table 1). Over the course of treatment, there were no elevations in hemoglobin, creatine, BUN (blood urea nitrogen) or insulin levels. There were 2 incidents of slightly and transiently elevated white blood counts and glucose levels, and 3 incidents of slightly and transiently elevated liver enzyme (ALT and AST) levels. All elevations resolved without interventions.

**Table 1:** Incidents of elevated blood values

	**#**	**Pre**^**6**^	**Mid**	**Post**
**WBC**^**1**^	2	0	1	1
**Glucose**^**2**^	2	1	1	0
**AST**^**3**^	3	0	2	1
**ALT**^**4,5**^	3	0	3	0

^1^White blood cell counts, normal range 3.8 to 10.5 × 1000 cells per mcl. Values of 11.0 and 12.0 recorded. ^2^Glucose, normal range 70–99 mg/dl. Values of 102 and 106 recorded. ^3^Aspartate aminotransferase, normal range 10–40 IU/L. Values of 42, 48, and 45 recorded. ^4^Alanine aminotransferase, normal range 10–45 IU/L. Values of 56, 60, and 48 recorded. ^5^ALT and AST elevations occurred in the same three patients. ^6^Pre, pre-trial; Mid, mid-trial; Post, post-trial.

**Figure 1:**
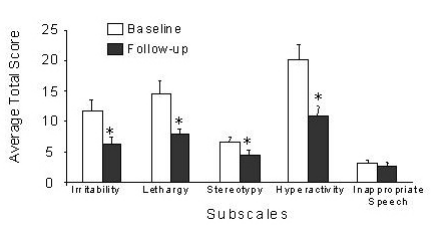
**Effect of Pioglitazone on behavior improvement**. The average (mean ± s.d.) of the total scores for the 5 subscales of the ABC was calculated for 25 patients before treatment (baseline) and after 3–4 months of treatment with Pioglitazone. *, P < .05 unpaired T-test.

A comparison of the mean scores for ABC subscales between baseline and end of treatment for each of the patients revealed that four of the five ABC subscales decreased significantly following the administration of pioglitazone (Figure 1). These subscales were hyperactivity, irritability, lethargy, and stereotypy. There was no change in inappropriate speech; however, it should be noted that the speech subscale is of limited value in children with autism who lack or have very limited speech.

Of the 25 patients, 76% showed an improvement (defined as >50% decrease in score) in at least one subgroup; while 56% showed an improvement in two or more subgroups, and 40% showed improvements in 3 or more subcategories. If response rate is estimated as those who showed >25% decrease in at least 2 of the 5 subscales, then the percentage is much higher 71%. The majority of patients (52%) showed an improvement (>50%) in the hyperactivity subscale.

**Figure 2:**
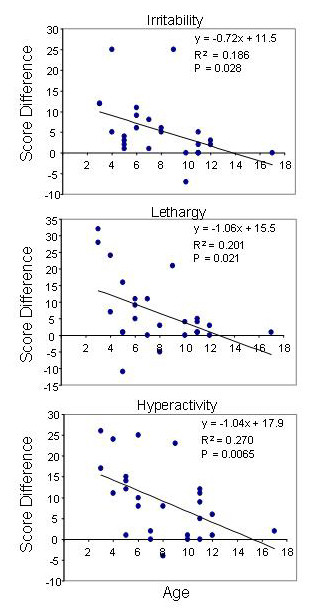
**Relationship of behavioral improvements to age**. Differences in scores for the 5 subscales of the ABC were calculated and plotted versus patient age, and analyzed using Graphpad Prism V4 assuming Gaussian distributions.

Significant inverse correlations (Figure 2) were detected between age and the improvements calculated for irritability (P = 0.03), lethargy (P = 0.02) and hyperactivity (P = 0.007). This indicates a tendency for younger participants to benefit more from pioglitazone than the older participants.

## Discussion and evaluation

The current study provides evidence that treatment with the PPARγ agonist pioglitazone (Actos) does not induce any significant adverse effects, and may have a beneficial effect on patterns of aberrant social behavior in children with diagnosed autism. Despite the small sample size (n = 25 total), we observed statistically significant decreases in 4 of the 5 subscales of the ABC after a relatively short (4 months) treatment with pioglitazone. It is yet not known if these improvements are long lasting, or if they will continue after treatment is withdrawn. Although originally approved for treatment of Type 2 diabetes in adults, recent clinical trials of pioglitazone for treatment of diabetes in adolescents suggest this drug will be well tolerated in younger populations [29,30].

There is increasing evidence for an association of ASD with various immune syndromes. It was reported that 66% of children with autism have a relative with an autoimmune disease [42], and families of children with PDD (Pervasive Development Disorder) have a higher average number of autoimmune diseases than families of healthy children [43]. Recently the occurrence of AITD (Autoimmune Thyroid Disease) in first or second order relatives was concluded to be a risk factor for those ASD children who show regression (the early loss of already established skills of communication or of social interactions) [44]. The possibility therefore exists that pioglitazone influences some aspect of auto-immune nature in ASD children.

It has been suggested that a Th2-like dysfunction may contribute to the causes of ASD. In children with ASD, a preponderance of Th2-like (IL4, IL6, IL10) over Th1-like (IL2, IFNg, IL1β) cytokines has been reported [45-48]. These studies support the idea that a predominance of Th2 cytokines may be a factor in ASD. PPARγ agonists are known to influence T-cell physiology, and although most often they have been shown to reduce Th1-like cytokine (IL1β, TNFa, IL12) production, in several studies they also reduced Th2 responses. In CD4 cells, PGJ2 and the TZD ciglitazone reduced IL4 production [35] and in EAE, the animal model of Multiple Sclerosis, PGJ2 blocked splenic T cell production of IL10 and IL4 [36]. PPARγ agonists also reduce the clinical symptoms in animal models of asthma, a disease which is also thought to be predominantly Th2 type involving IL4, IL5, and IL13 [37]. PPARγ agonists have been shown to reduce IL4, IL5, and IL13 production from Tcells of mice with induced lung inflammation [38,49]. However, in one study the TZDs increased IL4 and IL10, and stimulated GATA3 expression (a transcription factor which shifts cells towards Th2 phenotype) [50]; although in other studies PPARγ drugs were shown to inhibit GATA3 activity [51,52]. Nevertheless, taken together these studies demonstrate that PPARγ agonists have the potential to shift the T-cell response from Th2 to Th1, or to reduce Th2 cytokine expression, which may be of therapeutic benefit in ASD.

Despite observing significant improvements in 4 of 5 subscales of the ABC, the open-label nature of this study limits the ability to draw strong conclusions regarding treatment-dependent benefits. In addition, well-known expectancy effects in the parent population make interpretation of the ABC subject to potential bias [53,54]. The placebo effect in ASD has been reported to be high in some studies where improvement was assessed using the ABC. Improvements occurred in 25% of patients following atomoxetine treatment for 6 weeks, [55]; 34% after 8 week treatment with risperidone [56]; and 37% after 3 weeks treatment with amantadine [54]. In the current study, the number of responders (those showing >50% improvement in at least one subscale) was 76%, considerably higher than the values reported in the above studies.

An additional confound of the current study is the diversity of auto-immune comorbidities that are common in the autistic population. It is possible that pioglitazone effects are, in part or in full, an indirect consequence of reducing symptoms of the autoimmune diseases present in the study population (thyroiditis, colitis, and PANDAS). For example, in autoimmune thyroiditis (AITD), pioglitazone could increase levels of suppressor T-cells that are deficient [57] and as a result reduce circulating levels of Th1 or Th2 cytokines. Similarly, activation of PPARγ can suppress experimentally induced colitis [58] which could also reduce plasma cytokine levels, and in fact several clinical trials of PPARγ agonists for treating colitis are in progress [19,59]. PANDAS, a pediatric autoimmune neuropsychiatric disorder associated with streptococcal infections is defined by obsessive-compulsive (OCD) and or tic disorders, is thought to be due to the actions of auto-immune antibodies on basal ganglia neurons [60], and is improved by immunomodulatory therapies [61]; anti-inflammatory effects of PPARγ agonists could therefore influence the course of this disease. However, since the precise relationships between autoimmune diseases and the penetrance of autistic symptoms remains to be established, deciphering the relative importance of indirect effect of pioglitazone on behavior will be a formidable task.

The recent increase in type 2 diabetes in children has resulted in an increased interest of researchers to explore the use of anti-diabetic drugs including TZDs in children, therefore providing additional information regarding the safety of TZDs in this population. A recent clinical trial tested the effects of rosiglitazone (2 mg bid increased to 4 mg bid after 8 weeks), a related TZD, in 195 obese type 2 diabetic children (age range 8–17 years), in a 24-week double-blind, randomized, metformin-controlled, parallel group design. The rosiglitazone group gained ~3 kg after 24 weeks with the occurrence of peripheral edema in 1 child [29]. However, no other adverse effects were reported, suggesting that TZDs are well tolerated in children as in adults. More recently [30] pioglitazone (15 mg po escalated to 30 mg po after 4 weeks) was tested as an adjunct therapy for the treatment of type 1 diabetes in a small group of young adolescents (age range 10–17.9 years). After 6 months treatment the pioglitazone subjects showed a small but significant increase in BMI z-score (body mass index standard deviation for age) suggesting treatment-related weight gain. In the 35 subjects who completed the study, there was no evidence of edema, anemia, or of any significant increase in the frequency of hypoglycemia in the treatment group versus the placebo group. However, it is clear that the safety of pioglitazone, and of other TZDs, in the pediatric population requires additional testing.

## Conclusion

In view of its established safety profile, the current results provide the rationale for further testing of pioglitazone in autism and other forms of ASD.

## Abbreviations

ABC: Aberrant Behavior Checklist

AD: Alzheimer's disease

ASD: Autism Spectrum Disorder

BBB: Blood brain barrier

CBC: Complete blood count

CD: Cluster of differentiation

IL: Interleukin

MS: Multiple Sclerosis

NSAID: Non steroidal anti-inflammatory drug

PANDAS: Pediatric autoimmune neuropsychiatric disorder associated with streptococcal infections

PGJ2: 15-deoxy-delta12,14-prostaglandin J2

PDD: pervasive developmental disorder

PPAR: Peroxisome proliferator activated receptor

TNF: Tumor necrosis factor

TZD: thiazolidinedione

## Competing interests

The author(s) declare that they have no competing interests.

## Authors' contributions

MB and AG were the primary physicians who treated the patients, and carried out behavioral testing to determine if the medication was helping their patients. CK prepared the first draft of the paper, and analyzed the data. DLF organized and analyzed the data, contributed to the original idea to treat ASD patients, helped write and edit the manuscript.
